# Network analysis of *Mycoplasma pneumoniae* pneumonia and Epstein-Barr virus co-infection

**DOI:** 10.1186/s12879-026-13246-1

**Published:** 2026-04-10

**Authors:** Junya Song, Yin Lv, Xin Pei, Yan Wang, Shengnan Li, Yuehua Yao, Ning Jiang

**Affiliations:** Department of Pediatrics, Shandong Provincial Maternal and Child Health Care Hospital Affiliated to Qingdao University, Jinan, 250014 China

**Keywords:** *Mycoplasma pneumoniae*, *Mycoplasma pneumoniae* pneumonia, Epstein-Barr virus, Co-infection, Network analysis, Cytokines

## Abstract

**Background:**

*Mycoplasma pneumoniae*(*M. pneumoniae*) is one of the most frequent causes of community acquired pneumonia (CAP) in children, while Epstein-Barr virus(EBV) infection typically presents subclinical manifestations in immunocompetent children. Although single *M. pneumoniae* infection is common enough, *M. pneumoniae* and EBV co-infection has received little attention in clinical practice. It is worth noting that there have been reports of children with *Mycoplasma pneumoniae* pneumonia(MPP) coinfect with EBV often suffering from more severe lung injury and affecting multiple organ systems. However, the underlying shared targets or pathways remain unclear. This study aims to preliminarily explore the shared targets and pathways of EBV co-infection in pediatric patients with MPP by using network analysis methods.

**Methods:**

Relevant targets of MPP and EBV infection were obtained through database mining. The protein-protein interaction(PPI) network construction, Gene Ontology (GO) enrichment analysis and the Kyoto Encyclopedia of Genes and Genomes (KEGG) pathway enrichment analysis were employed to identify the shared core target genes and key signalling pathways.

**Results:**

A total of 425 MPP-related target genes and 744 EBV infection-related target genes were obtained, respectively. 113 shared targets were involved in co-infection, and 5 core proteins were obtained through screening, including tumor necrosis factor alpha(TNF-α), interleukin-6(IL-6), interleukin-2(IL-2), interleukin-10(IL-10), and interferon gamma(IFN-γ), which were common inflammatory cytokines. The clinical manifestations both inside and outside the lungs of MPP children with EBV co-infection were more severe than those of children with simple *M. pneumoniae* infection, which is related to the fact that their shared pathways are mainly enriched in immune responses.

**Conclusions:**

The results indicate that multiple shared inflammatory cytokines and pathways are present between M. pneumoniae and EBV co-infection, confirming that the severe symptoms and poor prognosis of co-infection are related to immune injury. As a network-based bioinformatics analysis, these findings are hypothesis-generating and require further experimental and clinical validation.

**Clinical trial number:**

Not applicable.

**Supplementary Information:**

The online version contains supplementary material available at 10.1186/s12879-026-13246-1.

## Introduction

*Mycoplasma pneumoniae* (*M. pneumoniae*) is one of the most important and common pathogens of community acquired pneumonia (CAP), accounting for approximately 10% to 40% of pediatric CAP cases [1–3]. Previous studies reported respiratory pathogens co-infection rates in children with CAP ranging from 12.2% to 35.3% [4–7]. Co-infection of *M. pneumoniae* is common in CAP [7–10]. For instance, Chi et al. demonstrated a high incidence (22.6%) of *M. pneumoniae* infection in Taiwanese children with CAP, among which 44.5% were coinfected with other pathogens, including *adenovirus* (ADV), *influenza A and B virus* (IVA and IVB), *rhinovirus* (RV), *respiratory syncytial virus* (RSV), etc [10]. Most cases of *Mycoplasma pneumoniae pneumonia (MPP)* are mild, and patients recover well. Some children had poor responses to conventional MPP treatment, presenting with persistent fever and worsening pulmonary imaging, which is called refractory *Mycoplasma pneumoniae* pneumonia (RMPP) [11]. The complex pathogenesis of RMPP remains unknown. In addition to factors such as fever duration, C-reactive protein (CRP), lactic dehydrogenase (LDH), and macrolide resistance, it may also be related to the co-infection with other pathogens [12–16]. However, previous studies mainly focused on clinical features, laboratory results, and treatments of MPP and RMPP, and there were relatively few reports on co-infection. Choo S et al. [15] reported that 43.4% MPP patients had respiratory virus co-infection. such as RV, ADV, RSV, and IV. Compared with the non-coinfection groups, longer durations of fever and hospitalization, elevated LDH and alanine aminotransferase (ALT), severe pneumonia, and poor response to the stepwise treatment were more common in the co-infection group (*P* < 0.05). These results suggest that pathogen co-infection might be a contributing factor for RMMP in children. Thus, studying the co-infection of MPP might have potential benefits for the early identification of RMPP and the timely use of appropriate drugs for the treatment of RMPP.

Besides *M. pneumoniae*, the known common pathogens in childhood CAP also include ADV, RV, RSV, IV, Epstein-Barr virus (EBV), *streptococcus pneumoniae* (SP), *staphylococcus aureus* (SA), etc [4, 5, 10, 16]. For children with MPP, co-infection of pathogens such as RSV and ADV is relatively common in clinical practice and is easy to detect. It has been reported that EBV DNA can be detected in the lungs of healthy subjects, as the respiratory tract is a major reservoir for EBV [17]. However, EBV infection usually presents as subclinical manifestations in immunocompetent children [18]. This clinical scenario is sometimes difficult to diagnose because it lacks unique symptoms. Therefore, co-infection of EBV and *M. pneumoniae* in the lung has received little attention. Xu et al. found that the detection rate of EBV co-infection in children with MPP was 53.7% [16]. It is worth noting that the co-infection group had a longer duration of fever, higher CRP, immunoglobulin A, immunoglobulin G, and interleukin-2 (IL-2) levels, higher peripheral neutrophil percentage, a higher incidence of pulmonary consolidation, and a higher percentage of RMPP, compared to the *M. pneumoniae* group. Similarly, the same conclusion was also identified in the research conducted by Hao et al. and Li et al. [19, 20]. These together indicated that, to some extent, MPP children with EBV co-infection had a more severe illness. Currently, the literature reports that M. pneumoniae and EBV co-infection can cause significant immune dysfunction, but the specific targets or pathways involved have rarely been described. Therefore, further research is necessary on these targets and pathways.

In recent years, network pharmacological analysis has been a research hotspot, primarily used to elucidate the complex correlations between drugs (especially multi-component drugs) and biological systems (diseases, targets, pathways). The principle was to identify the relevant disease targets and drug components, screen for intersection targets, then construct a multi-layer network of “drugs - components - targets - diseases”, and analyze and screen the key targets and pathways. It has been widely applied in research on the mechanism of traditional Chinese medicine compound prescriptions [21, 22]. Targets usually refer to the molecule through which a drug or active ingredient acts directly or indirectly in an organism. These molecules are involved in the occurrence and development of diseases and regulate related biological functions through interactions. Specifically, targets can be the following types of biological macromolecules: proteins (the vast majority), DNA, RNA, and lipids. Therefore, we used a network analysis method similar to network pharmacology to dig the disease targets of MPP and EBV infection respectively, and constructed a “disease – targets – disease” network to explore the **shared targets and pathways** involved in MPP and EBV co-infection.

## Methods

### Targets genes related to MPP and EBV infection

“ *Mycoplasma pneumoniae* pneumonia” and “Epstein-Barr virus infection” were used as search keywords. Data on the target genes related to MPP and EBV infection were mined from 4 databases: i) the Online Mendelian Inheritance in Man database(OMIM: https://www.omim.org/), an information platform mainly designed for human genes and genetic diseases. ii)the GeneCards database(https://www.genecards.org/), which provides comprehensive, user-friendly information on all annotated and predicted human genes. iii)the Genetic Association Database (GAD: http://geneticassociationdb.nih.gov/), which provides genes associated with complex diseases and disorders. iv) the DisGeNET database(https://www.disgenet.org/), which covers the full spectrum of human diseases. The obtained targets were deduplicated and standardized in the Uniprot database(https://www.uniprot.org/), a reference resource of protein sequences and functional annotation that covers proteins from all branches of the tree of life.

### Network construction and analysis

The intersection targets of MPP and EBV infection were extracted using Venny 2.1.0 (https://bioinfogp.cnb.csic.es/tools/venny/index.html), which may represent common targets of MPP and EBV co-infection. To study interactions among targets, the protein-protein interaction (PPI) network was constructed using the STRING platform (https://cn.string-db.org/, ver. 11.0), a database of known and predicted protein-protein interactions [23]. The species was set to “homo sapiens”, the minimum interaction threshold to “medium confidence 0.4”, and the rest of the parameters were kept at their default settings. Cytoscape software (https://cytoscape.org/, ver. 3.7.1) [24] was used to construct the visual network of MPP and EBV infection targets. The importance of each node in the network was evaluated using the topological parameters degree centrality (DC), betweenness centrality (BC), and closeness centrality (CC), which were calculated using CytoNCA, a Cytoscape plugin [25]. The DC of a node represents the number of nodes that directly interact with that node in the PPI network. The larger the DC value is, the more biological functions the node involved in, and the stronger its biological importance. The BC of a node refers to the proportion of the number of shortest paths in the network that pass through this node. The larger the BC value is, the more important the node is in the network. CC reflects the proximity of a given node to other nodes in the network. It is calculated as the reciprocal of the sum of the shortest path distances from a node to all other nodes. For a node, the closer it is to other nodes, the larger its CC will be, and the more important it is in the network. Based on the DC values, the size and color of the nodes are set, while the BC values are used to determine the line width. A PPI network diagram for screening core targets is then constructed. Finally, we selected “DC > average DC” as the primary parameter, then identified the shortest-path distance (CC) and shortest paths (BC) as the core targets [26].

### Enrichment analysis of GO and KEGG pathways

The Gene Ontology (GO, including biological process (BP), cell component (CC), and molecular functions (MF)) and the Kyoto Encyclopedia of Genes and Genomes (KEGG) pathway enrichment analysis were conducted for the common target genes of MPP-EBV co-infection using the Database for Annotation Visualization and Integrated Discovery (DAVID, https://davidbioinformatics.nih.gov/tools.jsp ), an online platform for the high-throughput functional annotation bioinformatics, to perform functional annotation and enrichment analysis [27]. Only GO terms with P-value < 0.05 [28] and KEGG pathways with P-value < 0.01 [29], FDR < 0.01 [30] are considered significant. Based on the above analysis, the main signaling pathways and biological processes of the co-acting targets will be evident, and the results will be visualized.

## Results

### Disease-related target genes

A total of 425 MPP-related target genes(Supplementary Table 1) and 744 EBV infection-related target genes (Supplementary Table 2) were obtained after deleting duplicate genes, respectively. 113 shared target genes were obtained by taking the intersection of the two target gene sets (Supplementary Table 3, Fig. 1).

### MPP – EBV infection PPI network construction and central network evaluation

To dive deeper into the interactions between targets, we first employed a topological method to evaluate the core network. The PPI network is beneficial for understanding the role of various proteins in complex diseases. A total of 113 nodes and 2271 edges were involved in the PPI network (Fig. 2). Larger DC values may be associated with larger nodes, and the color changed from green to red. The line width indicated the BC value. The thicker the line width, the larger the BC value. Among the 113 nodes (Supplementary Tables 4 − 1), 56 of them with DC values greater than the average DC value (Supplementary Tables 4 − 2), 31 nodes with BC and CC values greater than the average (Supplementary Tables 4 − 3). The top 5 proteins were identified as TNF-α, IL-6, IL-2, IL-10, and IFN-γ (Table 1).

**Table 1:** The top 5 protein topology parameters

Gene Name	Protein Name	DC	BC	CC
TNF-α	tumor necrosis factor alpha	92	0.0496267	0.84848485
IL-6	interleukin-6	88	0.03977093	0.81751825
IL-2	interleukin-2	85	0.0312364	0.8057554
IL-10	interleukin-10	83	0.0198215	0.79432624
IFN-γ	interferon gamma	79	0.02117225	0.76712329

DC: degree centrality; BC: betweenness centrality; CC: closeness centrality

### Enrichment analysis of the shared genes

According to a P-value < 0.05, 427 GO terms were identified. There were 342 items related to BP(Supplementary Tables 5 − 1), mainly involving immune response, inflammatory response, positive regulation of smooth muscle cell proliferation, regulation of apoptosis/proliferation, signal transduction, cell response to interleukin, T cell co-stimulation, innate immune response, cytokine chemotaxis, signal transduction, and response to glucocorticoids. There were 35 items on CC(Supplementary Tables 5 − 2), which mainly related to cytoplasm, cytoplasmic membrane, and extracellular region. There were 50 items related to MF(Supplementary Tables 5 − 3), mainly involving cytokine activity, protein binding, transcription factor binding, and receptor activity. The GO enrichment analysis diagram was made by selecting the top 15 items from the three aspects of BP, CC, and MF (Fig. 3).

KEGG pathway enrichment analysis was performed on the target genes, and 98 enrichment results(Supplementary Tables 6 − 1) were obtained. Based on *P* < 0.01 and FDR < 0.01, 77 major pathways (Supplementary Tables 6 − 2) were identified, and the results were visualized (Fig. 4). The size of the nodes in the bubble diagram represented the gene counts. The color indicated the P-value size. After excluding a wide range of signaling pathways, the main pathways were cytokine receptor pathway, Toll-like receptor (TLR) signaling pathway, tumor necrosis factor (TNF) signaling pathway, T cell receptor signaling pathway, EBV infection, NF-kappa B (NF-κB) signaling pathway, janus kinase signal transducer and activator of transcription (JAK-STAT) signaling pathway, NOD-like receptor (NLR) signaling pathway, B cell receptor signaling pathway, and chemokine signaling pathway. The top 20 major signaling pathways of *M. pneumoniae* with EBV co-infection were listed in Table 2.

**Table 2:** The major signaling pathways of *M. pneumoniae*/EBV co-infection

Term	KEGG pathway	Gene count	PValue
hsa04060	Cytokine-cytokine receptor interaction	34	7.04E-24
hsa05321	Inflammatory bowel disease (IBD)	21	3.45E-22
hsa05142	Chagas disease (American trypanosomiasis)	24	1.27E-21
hsa05140	Leishmaniasis	21	3.64E-21
hsa05330	Allograft rejection	17	1.34E-20
hsa05332	Graft-versus-host disease	16	8.93E-20
hsa05323	Rheumatoid arthritis	21	4.05E-19
hsa04620	Toll-like receptor signaling pathway	22	1.02E-18
hsa05168	Herpes simplex infection	26	4.27E-18
hsa04940	Type I diabetes mellitus	16	7.58E-18
hsa05164	Influenza A	25	1.75E-17
hsa04668	TNF signaling pathway	21	2.51E-17
hsa05152	Tuberculosis	25	2.63E-17
hsa05145	Toxoplasmosis	21	4.45E-17
hsa05144	Malaria	16	1.11E-16
hsa05320	Autoimmune thyroid disease	16	3.03E-16
hsa05161	Hepatitis B	22	9.56E-16
hsa04640	Hematopoietic cell lineage	18	3.71E-15
hsa05143	African trypanosomiasis	13	1.42E-14
hsa04660	T cell receptor signaling pathway	18	4.23E-14

## Discussion

This study used an intersection-target and PPI-based enrichment approach to explore potential shared biological processes. *M. pneumoniae* is not only a common pathogen of CAP in school-age and preschool children, but also the detection rate of *M. pneumoniae* in infants and young children aged 1 to 3 years has increased year by year [31–33]. After diagnosis, the disease can be effectively controlled by macrolide therapy. Patients with severe disease have complex clinical manifestations, which are associated with damage to multiple organs and systems, and then develop into severe or refractory MPP [11, 12, 14]. EBV infection is also more common in children. Most EBV-infected children are asymptomatic, and the virus can remain dormant for life after the initial infection [34]. The virus lurking in the child’s body may activate, causing injury to multiple organs and body systems [35, 36].

Although *M. pneumonia* and EBV are common pathogens among children, co-infection of *M. pneumoniae* and EBV in the respiratory tract has rarely been reported in immunocompetent patients and is not commonly recognized by clinicians. Some studies indicated that *M. pneumoniae* and EBV co-infection tend to have a poor prognosis [19, 20, 37, 38]. For instance, the report by Li et al. on a patient with M. pneumoniae and EBV co-infection who experienced splenic infarction [20], and the description by Yenson et al. of a 7-year-old with co-infection who had agglutination of leukocytes and erythrocytes [37]. Hao et al. conducted a retrospective analysis of clinical data from patients diagnosed with M. pneumoniae co-infected with EBV, those with isolated M. pneumoniae infection, and a control group of healthy youngsters [19]. They found that the fever duration in the co-infection group significantly exceeded that in the MP group (*P* < 0.05). Furthermore, Wang et al. detected the respiratory agents in children with infectious mononucleosis (IM) by indirect immunofluorescence [39]. The results indicate that 68.9% of children with IM had co-infection with multiple other pathogens. Among them, the incidence of *M. pneumoniae* co-infection was 38.0%. Meanwhile, fever, rash, lymphadenopathy, hepatomegaly, splenomegaly, atypical lymphocytes, and abnormal liver function were more frequent, and the length of hospital stay and duration of fever were longer than in a single infection. These reports further support the idea that co-infection with M. pneumoniae and EBV may be involved more severe disease manifestations. Further studies are needed to clarify the interaction process involved in co-infection by *M. pneumoniae* and EBV.

The major pathogenic mechanisms of *M. pneumoniae* infection mainly include adhesion to host cells, direct cytotoxicity against host cells, inflammatory response-induced immune injury, and immune evasion [40]. In its initial phase, *M. pneumoniae* attaches to the respiratory epithelial surface through surface adhesion proteins (P1, P30, P40, P90) [41, 42] and adhesion organelles. Attachment and invasion of *M. pneumoniae* produce direct damage to the host’s respiratory epithelium. This form of damage encompasses nutrient depletion, intracellular positioning, toxin discharge, oxidative harm, and the initiation of apoptosis [43]. In addition to adhesion injury and cytotoxicity, immune injury are associated with M. pneumoniae infection plays an important role in pathogenesis and is also a current research hotspot. After *M. pneumoniae* infection, its lipoproteins can be recognized by TLR1, TLR2, and TLR6, which stimulate the release of inflammatory cytokines including TNF-α, IL-2, IL-6, IFN-γ, and other inflammatory mediators [40]. Helper T cell 1 (Th1) cells mainly secrete pro-inflammatory cytokines such as TNF-α, IL-6, IL-2, and IFN-γ to enhance cytotoxicity. Th2 cells activate B lymphocytes to participate in humoral immune response by secreting anti-inflammatory factors such as IL-10, IL-4, and IL-5. The imbalance of Th1/Th2 cell differentiation was closely involved in the immunopathogenesis of MPP [44, 45]. EBV is a lymphocytic DNA virus of the Herpesvirus genus, and is mainly transmitted via saliva contact. The stages of the EBV life cycle include the primary infection, establishment of latency, and reactivation or lytic stage to produce new virions, and depend on the interplay between the virus and the host immune system [18]. EBV can infect human B lymphocytes through its target receptor CD21 to form lymphocyte recognition membrane antigens, which can activate CD4^+^T cells and CD8^+^T cells. With the expansion of natural killer (NK) cells and cytotoxic T lymphocytes (CTLs), infected cells are targeted and lysed. The virus in the cell is released into the blood and then infects new cells, thus achieving in-body transmission [46]. Patients infected with EBV can exhibit disorders in Th1/Th2 cytokine secretion. The large release of IL-6, IL-2, IFN-γ, and other factors can activate macrophages, which makes the negative feedback mechanism between CTL and antigen-presenting cells (APC) dysfunctional. Due to the cascade amplification effect, the appearance of cytokine storm may contribute to multi-system damage, such as IM, lymphoma, and hemophagocytic lymphohistiocytosis [47–49].

Studies have shown that immune dysfunction is very important in the pathogenesis of both *M. pneumoniae* and EBV infections. Therefore, clinical studies have emphasized that immune injury was the main reason why both *M. pneumoniae* and EBV infections are associated with multi-organ damage, and the co-infection had severe manifestations and a poor prognosis [16, 19, 20, 37, 38]. Previous studies have reported only some laboratory indicators following co-infection with *M. pneumoniae* and EBV, such as CRP, IL-2, IL-4, TNF-α,α and IL-12 [16, 19]. However, the specific targets or pathways involved in co-infections had not been reported. In cases of co-infections, an increase in the content of inflammatory cytokines is widespread, the simple detection of cytokines may have the drawback of low specificity in diagnosing pathogens. Currently, the diagnosis of EBV infection was based on the detection of specific antibodies against the EBV in the serum. The detection of some cytokines requires specialized reagents and equipments, but most grassroots hospitals do not have such conditions. Therefore, we employed network analysis methods to preliminarily explore the possible shared targets and pathways involved in *M. pneumoniae* and EBV co-infection for the first time. In our study, we found 113 shared target proteins involved in MPP and EBV co-infection, and a PPI network was constructed. A total of 5 core proteins were obtained through screening, including TNF-α, IL-6, IL-2, IL-10, and IFN-γ. They are all common inflammatory cytokines, which also suggests that the severe symptoms and poor prognosis of *M. pneumoniae* and EBV co-infection are indeed related to the immune injury.

Cytokines are among the most important effector and messenger molecules in the immune system. They profoundly participate in immune responses during infection and inflammation, protecting against or contributing to diseases such as allergy, autoimmunity, and cancer [44, 49, 50]. Tumor necrosis factor alpha (TNF-α) is a key mediator and regulator of immune responses; it governs the development of the immune system and cell survival signaling pathways [51]. Generally, an appropriate amount of TNF-α in the body can activate the immune system. However, excessive amounts can elicit local inflammatory responses, stimulate the secretion of other cytokines, and trigger a cascade effect, thereby aggravating the systemic inflammatory response and damaging the body’s tissues and organs [52]. Research showed that TNF-α levels increased in the peripheral blood of patients with MPP [53] and severe MPP(SMPP) [44] as well as those with RMPP [54]. Increased levels of TNF-α is better diagnostic ability for differentiation of RMPP with the best cut-off of 68.25pg/ml [54]. Furthermore, the high *M. pneumoniae*-DNA MPP group (BALF *M. pneumoniae*-DNA > 105 copies/mL) and SMPP groups had higher levels of IL-6, IL-10, and TNF-α in their BALF than the low *M. pneumoniae*-DNA MPP group (BALF *M. pneumoniae*-DNA ≤ 105 copies/mL) and mild MPP group(all *P* < 0 0.05) [55]. Elevated levels of TNF-α were also observed in patients with EBV infection [56, 57].

IL-6 is a pleiotropic cytokine involved not only in immune responses but also in inflammation, hematopoiesis, embryonic development and other fundamental processes [58], especially in cytokine storm [59]. Because IL-6 is an NF-κB target, simultaneous activation of NF-κB and signal transducer and activator of transcription 3 (STAT3) in non-immune cells triggers a positive feedback loop of NF-κB activation by the IL-6–STAT3 axis [60, 61]. The elevation of IL-6 reflects the intensity of the body’s inflammatory response to *M. pneumoniae* infection and is correlated with the degree of inflammatory infiltration and the extent of lung tissue damage. For instance, Gong et al. reported that IL-6 was one of the independent risk factors for SMPP, and established a diagnostic prediction model for SMPP with other three variables (ESR, PCT, and lung auscultation), which indicates excellent predictive performance [62]. Wang et al. found that elevated IL-6 (OR = 1.01; *P* < 0.001) in bronchoalveolar lavage fluid was an independent risk factor for MPP children complicated by plastic bronchitis [63]. Furthermore, Dong et al. showed that the inflammatory markers in children with MPP who had Multi-pathogen co-infections were significantly elevated (especially IL-6 and IL-10) [64]. Zhang et al. compared serum IL-6 levels between IM children and healthy children. The result revealed that the level of IL-6 was significantly higher than that of the healthy group(*P* < 0.05), and was related to the progression of disease [65]. Studies have shown that, after treatment, TNF-α and IL-6 levels in children with IM were significantly decreased [66]. Moreover, IL-6 expression was strongly detected in the stromal cells of nasopharyngeal carcinoma (NPC) related to EBV infection and in advanced-stage NPC patients compared to control subjects [67]. Therefore, IL-6 is considered a potential biomarker for assessing the severity and prognosis of MPP and EBV infection.

IL-2, as a T cell growth factor, plays an important part in the induction/suppression of immune responses [68]. IL-2 signaling activates the JAK-STAT, RAS-MAPK, and PI3K/AKT pathways, which modulate the expression, differentiation, proliferation, and cellular function of target genes [69]. Literature reports that IL-2 is involved in the immune response to MPP and EBV infections [16, 19, 65, 70, 71]. IL-2 > 57 pg/mL and IL-6 > 55.835 pg/mL are the diagnostic cut-off values for SMPP, with AUCs of 0.923 and 0.874, respectively, indicating high sensitivity and specificity [71]. Meanwhile, the level of IL-2 in the high EBV-DNA load group was also higher than that in the low EBV-DNA load group(*P* < 0.05), and the incidence of moderate to severe hepatomegaly and splenomegaly was higher in the high viral load group(*P* < 0.01, *P* < 0.05). Logistic regression study revealed that elevated blood IL-2 is an autonomous risk factor for worse prognosis in children with *M. pneumoniae* and EBV co-infection [16]. Additionally, ROC curve analysis suggested the predictive significance of serum IL-2 levels for undesirable outcomes. It’s worth noting that IL-2 therapy has been approved for the treatment of a variety of diseases, including oncology and autoimmune disease [72].

IL-10 family cytokines play essential roles in maintaining tissue homeostasis during infection and inflammation [73]. Multiple layers of signaling through the mitogen-activated protein kinase (MAPKs), extracellular signal-regulated kinase (ERK), and NF-kB pathways have been shown to be of importance in IL-10 gene regulation in myeloid cells [74]. Some teams reported significantly increased levels of IL-10 in MPP children [53, 70], which futher rose in RMPP children [48]. It is interesting to notice that the concentrations of TNF-α, IL-6, IL-1β, IL-4, IL-10 and IFN-γ increased significantly in MPP children than control group(children with foreign body aspiration), and further increased significantly in SMPP [70]. Li et al. made discoveries through monitoring the expression changes of cytokines in children with IM [75]. They found that IL-10 was one of good biomarkers for distinguishing IM patients from healthy controls. Huang et al. reported that, IL-6 and IL-10 were identified as as one of the two independent predictors for the progression from EBV-IM to Epstein-Barr virus-associated hemophagocytic lymphohistiocytosis (EBV-HLH) [76].

IFN-γ is mainly secreted by lymphocytes and can act on interferon receptors, exerting immunomodulatory effects by activating signal channels to express antiviral proteins. It is reported that, serum levels of IFN-γ were significantly increased in MPP [16, 70], SMPP [70] and RMPP children [48]. The research conducted by Dong et al. indicated that the level of IFN-γ in children with MPP had elevated, and this condition was more pronounced in severe cases [77], consistent with Peng et al. [78]. It was reported that children with acute IM had higher serum levels of IL-4, IL-6, IL-10, and IFN-γ than that of convalescent phase [79].

The above research indicates that, TNF-α, IL-6, IL-2, IL-10 and IFN-γ are shared inflammatory cytokines involved in MP and EBVco-infection. The clinical manifestations both inside and outside the lungs of MPP children with co-infection were more severe than those of children with simple infection, which is related to the fact that their shared pathways are mainly enriched in immune responses.

This study had significant limitations, Firstly, when mining targets through an online database, there are inevitably deficiencies. If the database is updated, it may have an impact on the analysis results. Secondly, the top 5 targets were listed, but no new molecular targets that might be helpful for diagnosing co-infection were identified. Lastly, we lack in vivo or in vitro experiments to validate our results. We had described and analyzed the specific shared targets of MPP and EBV infection based on previous clinical studies. Next, we plan to conduct further clinical research on the diagnostic value of shared targets. And conduct relevant experimental studies to explore the specific pathogenesis of these shared targets and pathways in the co-infection. As much as our results have identified common immune-related targets and pathways in MPP and EBV infection, the study is founded on network analysis with databases, instead of establishing a causal or clinical relationship. Propositions of clinical relevance of critical cytokines and pathways, therefore, must be regarded as hypothesis-generating. They need future clinical trials and experimental confirmation to identify their diagnostic, prognostic, or therapeutic usefulness.

## Conclusion

In our study, network analysis methods were used to explore the shared targets or pathways involved in *M. pneumoniae* and EBVco-infection for the first time. The results reveal that M. pneumoniae and EBV co-infection have multiple common inflammatory cytokines, including TNF-α, IL-6, IL-2, IL-10, and IFN-γ, which mainly focus on the inflammatory immune response. The excessive inflammatory response leads to severe organ damage. Clinically, for children with MPP, especially when they have a long fever duration, multiple organ involvement, significantly elevated inflammatory indicators, and progress to RMPP or SMPP, the possibility of EBV infection should be considered. Necessary EBV detection is conducive to early and correct clinical diagnosis and treatment. Meanwhile, for children with MPP complicated with EBV co-infection, the detection of TNF-α, IL-6, IL-2, IL-10, and IFN-γ will be helpful for evaluating the condition and prognosis. Overall, this network-based bioinformatics analysis found common targets and imposed enriched immune-related pathways in MPP and EBV infection. These results suggest biological connections and identify candidate molecules for further study. Nevertheless, the findings are speculative and require further experimental and clinical confirmation.

## Supplementary Information

Below is the link to the electronic supplementary material.

Supplementary Material 1: Supplementary Table 1: Known MPP targets. Supplementary Table 2: Known EBV infection targets. Supplementary Table 3: Common targets between MPP and EBV infection. Supplementary Table 4: Network centrality analysis and evaluation of significant targets for MPP and EBV infection. Supplementary Table 5: GO functional enrichment analysis. Supplementary Table 6: KEGG signaling pathways analysis

## Data Availability

Data is provided within the manuscript or supplementary information files.

## Abbreviations

*M. pneumoniae*: *Mycoplasma pneumoniae*
MPP: *Mycoplasma pneumoniae pneumonia*
EBV: Epstein-Barr virus
CAP: Community acquired pneumonia
RMPP: Refractory *Mycoplasma pneumoniae pneumonia*
SMPP: Severe *Mycoplasma pneumoniae pneumonia*
IM: Infectious mononucleosis
DC: Degree centrality
BC: Betweenness centrality
CC: Closeness centrality
PPI: Protein-protein interaction
CRP: C-reactive protein
LDH: Lactic dehydrogenase
ADV: Adenovirus
IVA and IVB: Influenza A and B virus
RSV: Respiratory syncytial virus
RV: Rhinovirus
SP: Streptococcus pneumoniae
SA: Staphylococcus aureus
TNF-α: Tumor necrosis factor alpha
IL-6: Interleukin-6
IL-2: Interleukin-2
IL-10: Interleukin-10
IFN-γ: Interferon gamma
